# Atomic mechanism of strong interactions at the graphene/sapphire interface

**DOI:** 10.1038/s41467-019-13023-6

**Published:** 2019-11-01

**Authors:** Zhipeng Dou, Zhaolong Chen, Ning Li, Shenyuan Yang, Zhiwei Yu, Yuanwei Sun, Yuehui Li, Bingyao Liu, Qiang Luo, Tianbao Ma, Lei Liao, Zhongfan Liu, Peng Gao

**Affiliations:** 1 Electron Microscopy Laboratory, School of Physics, Peking University, Beijing, 100871 China; 2 Key Laboratory for Micro-/Nano-Optoelectronic Devices of Ministry of Education, School of Physics and Electronics, Hunan University, Changsha, 410082 China; 3 Center for Nanochemistry (CNC), Beijing Science and Engineering Center for Nanocarbons, College of Chemistry and Molecular Engineering, Peking University, Beijing, 100871 China; 4 International Center for Quantum Materials, Peking University, Beijing, 100871 China; 5 State Key Laboratory of Superlattices and Microstructures, Institute of Semiconductors, Chinese Academy of Sciences, Beijing, 100083 China; 6 Center of Materials Science and Optoelectronics Engineering, University of Chinese Academy of Science, Beijing, 100049 China; 7 State Key Laboratory of Tribology, Tsinghua University, Beijing, 100084 China; 8 Beijing Graphene Institute (BGI), Beijing, 100095 China; 9 Collaborative Innovation Center of Quantum Matter, Beijing, 100871 China; 10 Beijing Key Laboratory of Quantum Devices, Beijing, 100871 China

**Keywords:** Graphene, Two-dimensional materials, Surfaces, interfaces and thin films

## Abstract

For atomically thin two-dimensional materials, interfacial effects may dominate the entire response of devices, because most of the atoms are in the interface/surface. Graphene/sapphire has great application in electronic devices and semiconductor thin-film growth, but the nature of this interface is largely unknown. Here we find that the sapphire surface has a strong interaction with some of the carbon atoms in graphene to form a C-O-Al configuration, indicating that the interface interaction is no longer a simple van der Waals interaction. In addition, the structural relaxation of sapphire near the interface is significantly suppressed and very different from that of a bare sapphire surface. Such an interfacial C-O-Al bond is formed during graphene growth at high temperature. Our study provides valuable insights into understanding the electronic structures of graphene on sapphire and remote control of epitaxy growth of thin films by using a graphene–sapphire substrate.

## Abstract (web summary)

Understanding the atomic arrangement and binding nature of 2D materials with substrates is crucial to understand their properties and utilize their functions. Here, authors report that at high temperature graphene and α-Al_2_O_3_ substrate form a C-O-Al bond, having strong interactions, while interfacial structural relaxations of sapphire remain suppressed.

## Introduction

The interface is the device^1^. At the interface of crystal materials, the broken translation symmetry usually strongly alters the optical, electric, and magnetic properties, resulting in many interesting phenomena such as interface-induced superconductivity^2,3^, two-dimensional (2D) electron gas^4^, and magnetoelectric coupling^5,6^. In particular, the interface effects are much more pronounced or dominant in atomically thin 2D materials (e.g., graphene (Gr)), because most of the atoms are part of the interface when supported on a substrate. For instance, in a field-effect transistor system, strong hybridization (orbital overlap) between 2D materials and contact metals can eliminate the van der Waals (vdW) gap, which results in a reduction of the tunnel barrier for carriers^7^. Such an effect is also observed in 2D/2D heterostructures, e.g., the topological current is detected in the Gr/*h*-BN superlattice^8^ and the reconstruction at the Gr/*h*-BN interface provides a periodic scattering potential for carriers, leading to very unique electrical properties^9^. Moreover, coupling of the Gr/Gr interface via the “magic” angle results in correlated insulating states at half-filling^10^ and can be tuned to zero-resistance states upon electrostatic doping^11^.

Recently, Gr/sapphire (α-Al_2_O_3_) has shown promising applications in electronic devices due to the relatively high dielectric properties of α-Al_2_O_3_ as a gate dielectric^12,13^. In addition, Gr/α-Al_2_O_3_ is also applied as a platform for remote growth of third-generation semiconductors where Gr acts as a buffer layer to overcome the limitation of lattice mismatch^14–16^. The penetration of the electrostatic potential from substrates is governed by the interface nature, which eventually determines the epitaxial crystal quality^17,18^. Therefore, it is vital to reveal the atomic structure and binding nature of the Gr/α-Al_2_O_3_ interface^19,20^. Although considerable advances have been achieved in characterization techniques, little is known about the structure and properties of the Gr/α-Al_2_O_3_ system because of the lack of effective methods to probe the atomically thin interface of Gr on insulating substrates. For instance, the common scanning tunneling microscopy and low-energy electron diffraction (LEED) methods are usually restricted to conductive substrates, but α-Al_2_O_3_ is nonconductive. Therefore, most of the previous knowledge is obtained from computational approaches such as density functional theory (DFT) calculations^21^. Aberration-corrected transmission electron microscopy (Cs-TEM), which enables atomically resolved identification of the structure and chemical composition of materials regardless of their electric conductivity, provides alternative opportunities to study the atomic structure and properties of the interface and surface for insulators^22,23^.

Here, by Cs-TEM, we successfully reveal the atomic arrangement of the Gr/α-Al_2_O_3_ interface and accurately measure the gap between Gr and α-Al_2_O_3,_ which governs the interaction between the substrate and functional layers. We observe that the atomic configuration at the interface is C-O-Al, which is further confirmed by DFT calculations. Such an interfacial bond induces a large adhesion energy of ~1.47 J/m^2^, indicating that the interaction of the Gr/α-Al_2_O_3_ interface is no longer a simple vdW interaction. In addition, we also precisely obtain the structural relaxation of the surface layers of α-Al_2_O_3_ and find that the structure of Al_2_O_3_ near the interface is significantly different than either the bulk or bare α-Al_2_O_3_ surface. The contraction rate of the first Al layer at the interface is −(35.3 ± 8.2)% with respect to the bulk value compared with −79.4% for a bare α-Al_2_O_3_ surface. The pristine surface of α-Al_2_O_3_ is further identified to be Al-terminated and the interfacial C-O-Al bond is likely formed during high-temperature growth of Gr. All these findings provide valuable insights into the binding nature between 2D materials on a substrate and would be beneficial to the strategy of remote epitaxial growth by using a Gr buffer layer.

## Results

### Characterization of the Gr/α-Al_2_O_3_ interface

To ensure the cleanness of the interface, we directly grew a Gr film on α-Al_2_O_3_ substrates via a chemical vapor deposition (CVD) method (see details in the Methods section). For better protection of the Gr/α-Al_2_O_3_ interface, a multilayer Gr film was obtained by prolonging the growth time. Raman mappings of *I*_G_ and *I*_2D_ of the obtained Gr/α-Al_2_O_3_ in Supplementary Fig. 1a, b show a high uniform color contrast at the microscale, indicating the uniformity of the grown Gr film. The relatively low value of *I*_2D_/*I*_G_ (≤0.8) confirms the multilayer feature of Gr (Supplementary Fig. 1c). Moreover, the G and 2D peaks of CVD-derived Gr/α-Al_2_O_3_ are all upshifted with respect to those of transferred Gr on SiO_2_/Si substrates (Supplementary Fig. 1d), indicating that Gr experiences a compressive strain imposed by α-Al_2_O_3_.

**Fig. 1:**
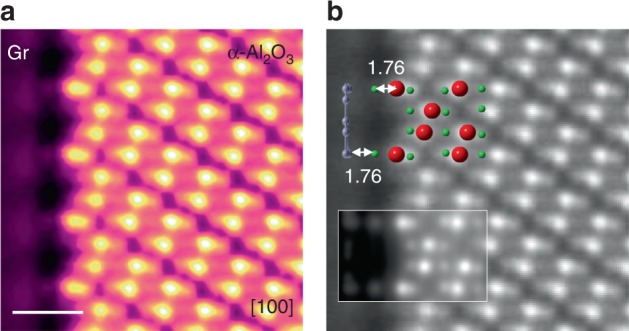
Atomic structure of the Gr/α-Al_2_O_3_ interface. **a** HAADF image viewing along the [100] orientation. The image is colored to clearly show the interface structure. **b** The corresponding ball and stick model and simulated image. The unit of length is Å. Scale bar: 0.5 nm. Red: Al atom. Green: O atom. Gray: C atom

We used the high-angle annular dark-field scanning TEM (HAADF-STEM) imaging mode of Cs-TEM to reveal the atomic structure of the Gr/α-Al_2_O_3_ interface. The atomically resolved image of the Gr/α-Al_2_O_3_ interface with a viewing direction of [100] is shown in Fig. 1a and the Gr layer can be clearly observed in an image with lower magnification in Supplementary Fig. 2. The corresponding atomic structures of α-Al_2_O_3_ and Gr are depicted in Fig. 1b from the [100] direction superimposed on the STEM image and are in good agreement with the simulated result. We observe additional atoms between the Gr layer and the topmost surface Al atoms. These atoms are further identified to be O, which will be explained later in detail. The distance between the topmost surface Al and the additional O is ~1.76 Å, whereas the spacing between the O and the Gr is also ~1.76 Å, indicating a strong interaction between Gr and the α-Al_2_O_3_ substrate.

The presence of the C–O bond at the interface is further confirmed by the C 1*s* X-ray photoelectron spectroscopy spectrum (Supplementary Fig. 3) of Gr/α-Al_2_O_3,_ which shows an intense *sp*^2^ carbon peak (~284.8 eV), a weak C–H peak (~285.7 eV), and a weak C–O peak (~287.3 eV). Such a C–O peak suggests the existence of a C–O–Al configuration at the interface^24^.

### DFT calculation of the Gr/α-Al_2_O_3_ interface

**Fig. 2:**
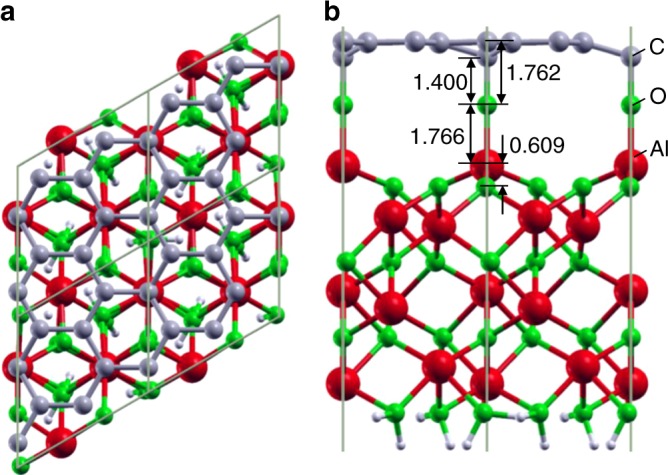
Calculated interface structure of Gr/α-Al_2_O_3_. **a** Top view. **b** Side view. The unit of length is Å. Red: Al atom. Green: O atom. Gray: C atom

We perform DFT calculations to verify the Gr/α-Al_2_O_3_ interface structures^21^. To construct the Gr/α-Al_2_O_3_ interface, we first added an additional atom on top of the surface Al atom according to the STEM results and then added the Gr layer (Supplementary Methods for details). We consider all the possibilities (O, H, C, and Al) for the additional atoms and find that only the O atoms can produce an interface structure consistent with the experimental results, as shown in Supplementary Figs. 4–6. The structure without additional atoms at the interface is also shown in Supplementary Fig. 7 for comparison. Figure 2a,b shows the top and side views, respectively, of the geometry of the Gr/α-Al_2_O_3_ interface with the additional O atoms obtained from DFT calculations. The additional O atoms directly bond to some of the C atoms of Gr with a bond length of 1.400 Å, as shown in Fig. 2b, whereas the other C atoms are further away from the α-Al_2_O_3_ surface. As a result, the average spacing between the additional O atoms and the Gr is 1.762 Å, which is in good agreement with the experimental results.

### Effects on the surface structure of α-Al_2_O_3_

**Fig. 3:**
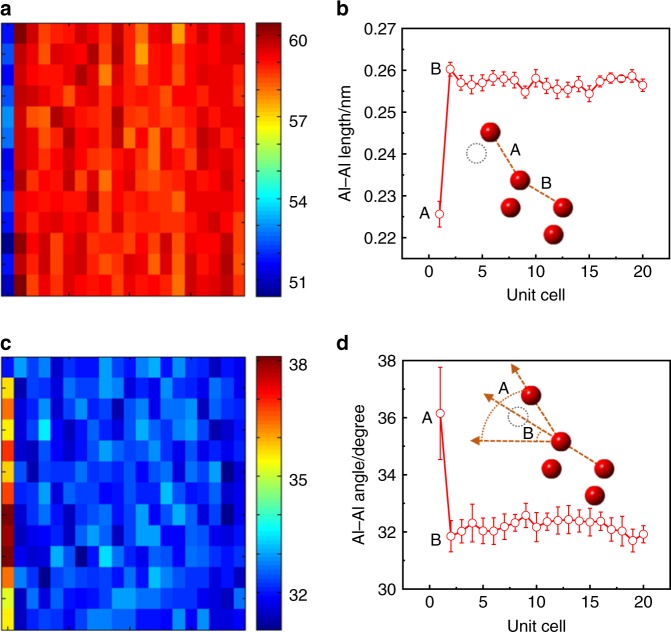
Quantitative measurements of Al–Al length and angle. **a** Al–Al length mapping. **b** Averaged distance. **c** Al–Al angle mapping. **d** Averaged angle. The error bars are the  standard deviation (SD)

Figure 3 shows the quantitative analysis of the surface structure of α-Al_2_O_3_ along the [100] orientation based on the image shown in Fig. 1a. We measured the distance between Al atoms from different layers (Al–Al length) and the angle between the Al–Al direction and the normal direction of the interface (Al–Al angle). There are negligible deviations of the Al–Al distance and angle far below the surface compared with bulk α-Al_2_O_3_, whereas a significant difference is observed for the top surface layers, as shown in Fig. 3a,c. In our experiment, the Al–Al distance at the surface is ~2.3 Å, which is in sharp contrast to the bulk value of ~2.6 Å. Furthermore, a similar trend is observed for the Al–Al angles, i.e., the angle at the surface is ~36°, whereas it is ~32.5° in the bulk. The DFT calculations also show a large change in surface relaxation for α-Al_2_O_3_, i.e., 2.22 Å for the Al–Al distance at the surface and 2.58 Å inside the bulk and 38.64° for the Al–Al angle at the surface and 32.39° inside the bulk, which is in excellent agreement with the experimental results.

**Fig. 4:**
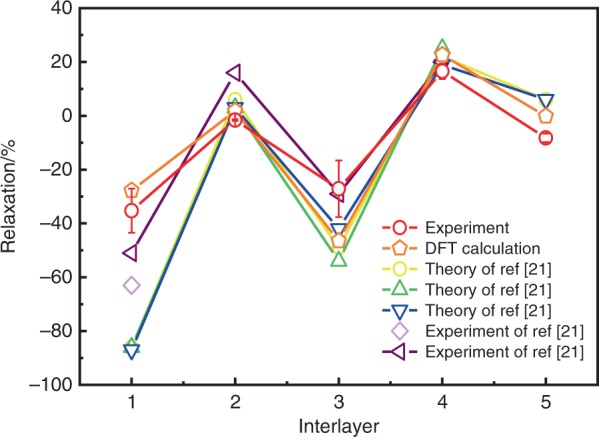
Interlayer relaxation of α-Al_2_O_3_ in percent of the corresponding bulk spacings. The relaxation from the first layer (Al) to the second layer (O) is −(35.3 ± 8.2)%. The DFT calculation and previous results are also plotted for comparison. The error bar of experimental relaxation is calculated from the SD of bond length

Indeed, the structure relaxation at the surface is an important physical phenomenon in many materials^25–27^, including α-Al_2_O_3_. We now compare the structure relaxation of α-Al_2_O_3_ near the interface with the bare surface case reported in the literatures in Fig. 4. In our case, the surface contraction is approximately −(35.3 ± 8.2)% for the top layer (distance from the first Al layer to the second O layer) compared with the bulk, whereas the relaxation rates become −(1.6 ± 0.3)%, −(27.1 ± 10.5)%, (16.6 ± 2.9)%, and −(8.2 ± 1.6)% for the second, third, fourth, and fifth layers, respectively. These measured values are very close to our DFT calculations and typically smaller than that of a bare α-Al_2_O_3_ surface reported in previous DFT calculations and experiments^21^, indicating that the strong interaction between the α-Al_2_O_3_ surface and the C atoms in Gr effectively suppresses the structure relaxation of the α-Al_2_O_3_ surface.

## Discussion

Thus far, we have obtained the atomic structure of the Gr/α-Al_2_O_3_ interface and identified the C–O–Al bonding at the interface. It is a prerequisite to determine the pristine structure of the α-Al_2_O_3_ surface before growth of Gr to understand the formation of the C–O–Al interfacial structure. In fact, much effort has been made to reveal the surface structure of α-Al_2_O_3_, because it is widely used as a substrate for the growth of thin metals, high-temperature superconductors, 2D materials, and semiconductor films^28–30^. Nevertheless, there are controversies regarding the termination layer of α-Al_2_O_3_. Both empirical and theoretical simulations identified only one stable Al-termination stoichiometry for the α-Al_2_O_3_ surface, whereas a few studies reported a stable O-termination structure^21^. A recent study of LEED measurements reported that the ratio of Al- to O-terminated domains is 2:1^31^.

We attempt to directly reveal the surface structure of pristine α-Al_2_O_3_ using the same Cs-TEM method, however, during the TEM specimen thinning process, the surface amorphization became severe, preventing us from identifying the termination layer of the pristine α-Al_2_O_3_ surface. We then transferred Gr onto α-Al_2_O_3_ to avoid any high-temperature process before the sample thinning process to protect the α-Al_2_O_3_ surface. We find that no bridging O atom is observed at the interface of the transferred Gr/α-Al_2_O_3_, and that the surface of α-Al_2_O_3_ is Al-terminated in the ambient environment, as shown in Supplementary Fig. 8a. We also perform DFT calculations, which further confirm that the Al-termination layer is the most stable for the α-Al_2_O_3_ surface in Supplementary Fig. 9.

Therefore, it seems that during high-temperature growth of Gr, the O atoms in the α-Al_2_O_3_ substrate escape to the interface and are captured by the C atoms to form the C–O–Al configuration. To further verify the role of high temperature, we subsequently annealed the transferred Gr on α-Al_2_O_3_ at 1050 °C. We find that such an interfacial C–O–Al configuration can also be observed in this case (Supplementary Fig. 8b), further confirming that the C–O bond is formed at high temperature.

The formation of C–O–Al bonds can significantly change the properties of the interface. The adhesion force/energy of the Gr/α-Al_2_O_3_ interface is evaluated using a mechanical nanoscratch method as shown in Supplementary Fig. 10^24^. The interfacial interaction energy of directly grown Gr on α-Al_2_O_3_ is ~1.47 J m^−2^, which is in excellent agreement with our DFT calculations (1.40 J m^−2^). In comparison, the interaction energy of the transferred Gr/α-Al_2_O_3_ interface is only ~0.66 J m^−2^, which is close to the DFT calculation value of 0.46 J m^−2^. After high-temperature annealing, the interaction energy of the transferred Gr increases to ~1.25 J m^−2^, further confirming the formation of a strong interface bond between Gr and α-Al_2_O_3_ at high temperature.

Such a strong chemical interaction between Gr and α-Al_2_O_3_ completely contrasts with the common assumption of vdW interaction^32,33^. This result provides valuable insight into the remote epitaxy of single-crystalline materials on Gr-coated substrates. The strong interaction between Gr and the substrate (herein, C–O–Al) enables relatively strong atomic interactions from the underlying substrates to penetrate Gr. However, the force between Gr and the epilayer is reduced by nearly two orders of magnitude compared with the covalent bonds formed in the conventional epitaxy approach due to the vdW epitaxy feature^17^. In addition, the strong interaction between Gr/α-Al_2_O_3_ would create a modulated potential that breaks the symmetry, changes the on-site energy of atoms, and opens up a small band gap around the K-point^34,35^, which provides promising opportunities for next-generation electronics.

In summary, we obtained the interface structure of Gr/α-Al_2_O_3_ by Cs-TEM. We find that the topmost layer is a reconstructed O layer, which directly bonds to Gr to form the C–O–Al interface configuration. In addition, with the presence of Gr and interfacial bonding, the structure relaxation behavior of α-Al_2_O_3_ near the interface is suppressed compared with that of the bare surface. All these results are in good agreement with the DFT calculations. Our study suggests that the interface of Gr/α-Al_2_O_3_ is not as simple as assumed and the interaction between Gr and substrate is a nonpure vdW interaction. The formation of strong bonding between Gr and α-Al_2_O_3_ is expected to significantly change the optical, electrical, and mechanical properties of Gr. In particular, it provides valuable insights into the remote control of the epitaxy growth of thin films by using Gr as a buffer layer.

## Methods

### Gr synthesis and transfer

Typically, the commercial α-Al_2_O_3_ substrate (purchased from Unionlight Technology, Co., Ltd) was loaded into a three-zone high-temperature furnace after the cleaning process. The furnace was heated to 1050 °C and was stabilized for ~10 min under 100 s.c.c.m. Ar and 100 s.c.c.m. H_2_, and 20 s.c.c.m. CH_4_ was introduced into the reaction chamber as a carbon source for the growth of Gr on the α-Al_2_O_3_ substrate for ~3–5 h.

### Transfer of Gr on α-Al_2_O_3_

The CVD grown Gr on copper is also transferred onto α-Al_2_O_3_ for comparison, in which process poly (methyl methacrylate) was used as the polymer support^36^.

### Annealing of the transferred sample

The transferred sample was loaded into a high-temperature furnace and heated to 1050 °C, and then annealed for ~10 min under the protection of 500 s.c.c.m. Ar, 100 s.c.c.m. H_2_.

### TEM sample preparation

The Gr/α-Al_2_O_3_ TEM specimen was prepared by mechanical polishing followed by argon ion milling (Gatan Model 691 system) with a gun voltage of 1.5–4.5 kV (beam angle of ~1.5–6°). At the final stage of ion milling, the voltage was set at 1.5 KV for ~3 min to remove the surface amorphous layer and minimize the damage.

### Adhesion force/energy measurement

The adhesion force/energy of the Gr film on α-Al_2_O_3_ was determined using a mechanical nanoscratch method with the aid of an AFM system (Cypher Asylum Research). The radius of the diamond tip used for the measurement was ~40 nm and a normal load of 11.9 μN was applied.

### Data acquisition and analysis

The atomic scale HAADF-STEM images were acquired using a double aberration-corrected FEI (Titan Cubed Themis G2, spatial resolution up to 60 pm) operated at 300 kV with the convergence semiangle 21.5 mrad and collection semiangle snap 80–379 mrad. We also deliberately minimized the electron dose to avoid specimen damage using a small aperture, small beam current, and short scanning time. The atomistic models were generated by VESTA and XCrysDen software. The plots were created by Origin 2018C. Atom positions were determined by simultaneously fitting 2D Gaussian peaks to an a priori perovskite unit cell using a home-developed code running in MATLAB R2012b. The data mapping was also carried out by MATLAB R2012b.

### HAADF-STEM image simulation

The multislice STEM simulation was performed using the software pack developed by Kirkland^37^. We used the single layer Gr/α-Al_2_O_3_ interface model shown in Fig. 2. In the simulation, the wave function size, sampling size, and image size were set to 1024 × 1024, 512 × 512, and 512 × 512, respectively. All microscope settings were the same as those in the experiments. The simulated image was then convolved with an 80 pm full width at half maximum  Gaussian function considering the incoherent source broadening.

## Supplementary information

Supplementary Information

## Data Availability

All data supporting the findings of this study are available within the paper and its Supplementary Information.
