# Supplementary material for: Atomic mechanism of strong interactions at the graphene/sapphire interface
Source: Nat Commun. 2019 Nov 1;10:5013. doi: 10.1038/s41467-019-13023-6 (PMC6825119; doi:10.1038/s41467-019-13023-6)
Supplement: Supplementary file 1 — Supplementary Information [file 41467_2019_13023_MOESM1_ESM.pdf]

Supplementary Information

Atomic Mechanism of Strong Interactions at the Graphene/Sapphire Interface

Zhipeng Dou[1,2#], Zhaolong Chen[3#], Ning Li[1,4#], Shenyuan Yang[5,6]*, Zhiwei Yu[7], Yuanwei Sun[1,4], Yuehui Li[1,4], Bingyao Liu[1], Qiang Luo[4], Tianbao Ma[7], Lei Liao[2]*, Zhongfan Liu[3,8], and Peng Gao[1,4,9,10]*

[1]Electron microscopy laboratory, School of Physics, Peking University, Beijing 100871, China

[2]Key Laboratory for Micro-/Nano-Optoelectronic Devices of Ministry of Education, School of Physics and Electronics, Hunan University, Changsha 410082, China

[3]Center for Nanochemistry (CNC), Beijing Science and Engineering Center for Nanocarbons, College of Chemistry and Molecular Engineering, Peking University, Beijing 100871, China

[4]International Center for Quantum Materials, Peking University, Beijing 100871, China

[5]State Key Laboratory of Superlattices and Microstructures, Institute of Semiconductors, Chinese Academy of Sciences, Beijing 100083, China

[6]Center of Materials Science and Optoelectronics Engineering, University of Chinese Academy of Science, Beijing 100049, China

[7]State Key Laboratory of Tribology, Tsinghua University, Beijing 100084, P. R. China

[8]Beijing Graphene Institute (BGI), Beijing, 100095, P. R. China

[9]Collaborative Innovation Center of Quantum Matter, Beijing 100871, China

[10]Beijing Key Laboratory of Quantum Devices, Beijing 100871, China

*Correspondence: syyang@semi.ac.cn; liaolei@whu.edu.cn; p-gao@pku.edu.cn;
[#] These authors contributed equally to this work.

Supplementary Figures 1-10

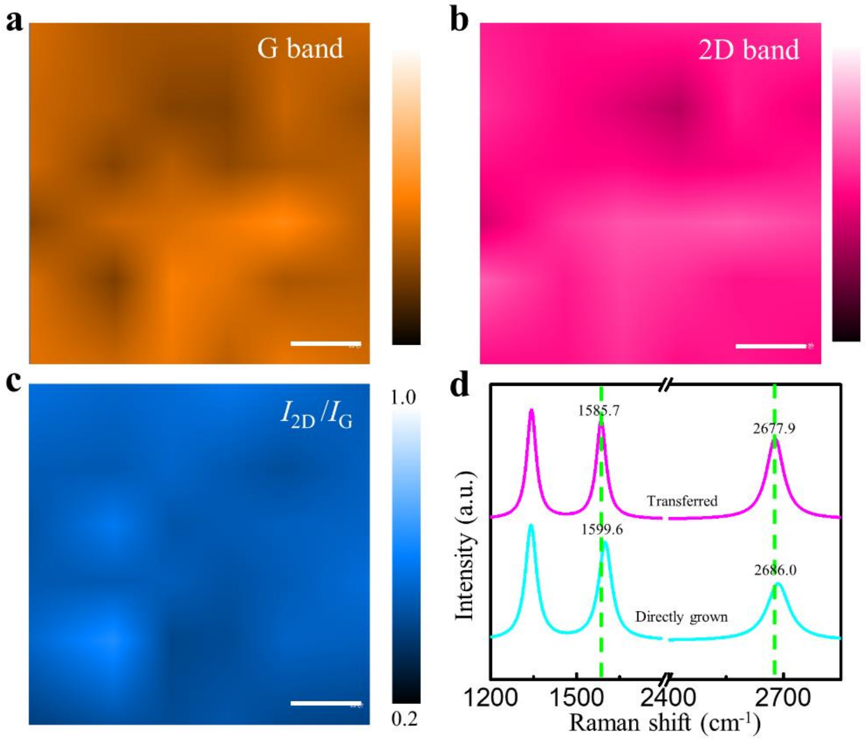

Supplementary Figure 1. Raman mapping and spectra of the directly grown graphene (Gr) on sapphire (α-Al_2O_3). (a) G peak. (b) 2D peak. (c) I_{2D}/I_{G}. (d) Raman spectra of directly grown Gr on α-Al_2O_3 and transferred Gr onto SiO_2/Si substrate. Scale bar: 1 μm.

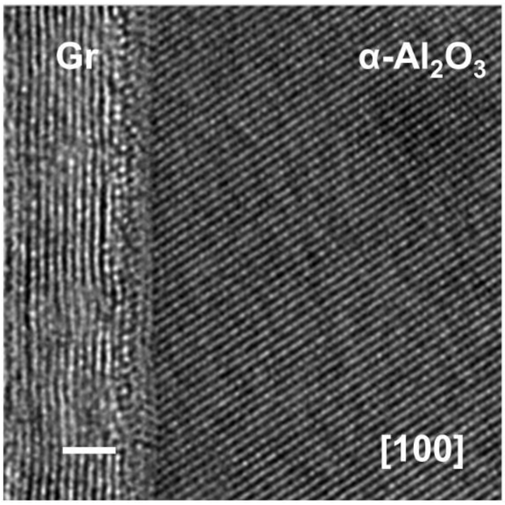

Supplementary Figure 2. Low magnification TEM of the interface of Gr on α-Al_2O_3.
Scale bar: 2 nm.

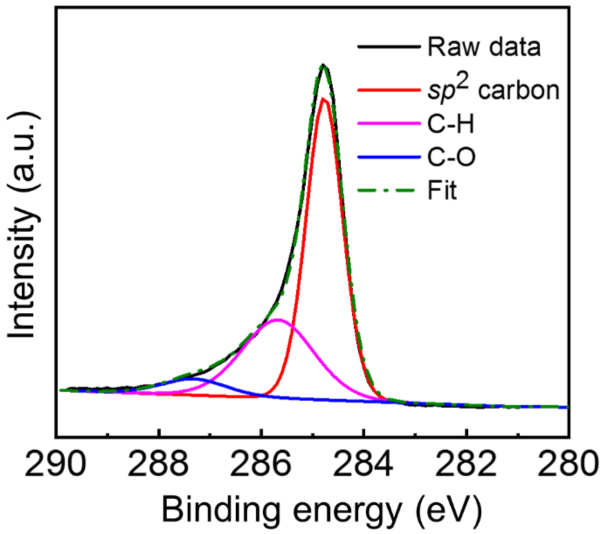

Supplementary Figure 3. The C *1s* X-ray photoelectron spectroscopy (XPS) spectrum of Gr/α-Al_2O_3 showing an sp^2 carbon peak (~284.8 eV), a C-H peak (~285.7 eV) and a C-O peak (~287.3 eV).

Supplementary Methods. The first-principles calculations based on DFT are carried out using the Vienna ab-initio simulation package (VASP)[1] with the generalized-gradient approximation of Perdew, Burke, and Ernzerhof (PBE)[2] for the exchange correlation functional. We adopt the all-electron-like projector-augmented wave potentials[3] and the energy cutoff for the plane-wave expansion was set as 400 eV. To model the interface, the Gr 2×2 unit cell is compressed by 2.75 % to match the α-Al_2O_3(0001) 1×1 surface cell. The Al-terminated α-Al_2O_3(0001) surface is modeled by an eight-layer slab with the bottom passivated by pseudo-hydrogen atoms. These pseudo-hydrogen atoms and the three bottom layers of α-Al_2O_3 are kept fixed during the relaxation. The other atoms are fully relaxed until the Hellmann-Feynman forces on each atom are less than 0.01 eV/Å. A vacuum larger than 15 Å is included to ensure a negligible interaction between images. Monkhorst-Pack k-point mesh[4] for the Brillouin zone sampling is set as 9×9×1. Weak van der Waals (vdW) interactions are included with the Becke88 optimization (optB88) functional[5].

To identify the additional atoms between the α-Al_2O_3 and Gr observed in TEM measurements, we consider all the possible atoms in our DFT calculations, including O, H, C, and Al atoms. We also shift the Gr layer in-plane to get the most stable interface structures, where one C atom from Gr is directly bonded to the additional atom, as shown in Figs.2 and Supplementary Figures 4-6. For O atom, the interface structure is in good agreement with the TEM measurements, as discussed in the paper. However, for H, C, and Al atoms, the interface structures from DFT calculations have distinct difference from the TEM measurements. The distances from the additional atom to the surface Al atom and to the Gr layer do not simultaneously match the TEM results. Especially, the interlayer distance between the surface Al and the subsurface O layers is much smaller than the TEM result. This can be attributed to the weaker binding between the surface Al atom and the additional atom, and thus the surface Al atom has a larger surface contraction.

For comparison, we also calculate the Gr/α-Al_2O_3 interface without additional atoms between the surface Al atom to Gr layer. As shown in Supplementary Figure 7, the distance between the surface Al atom to Gr layer is about 2.8 Å due to a weak

interaction between Al and C atoms. Consequently, the interlayer distance between the surface Al and the subsurface O layers is only 0.23 Å, similar to the clean surface. Obviously, such structure is not consistent with the TEM images.

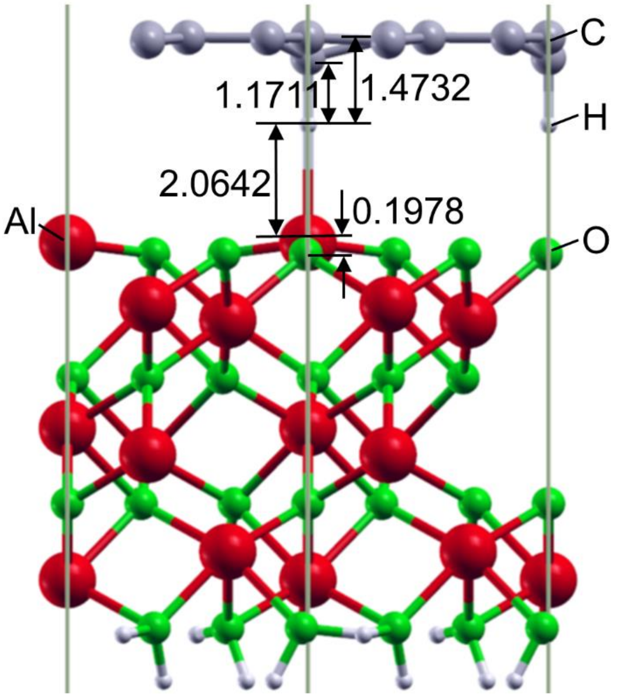

Supplementary Figure 4. DFT calculation of Gr/α-Al_2O_3 with H at the interface. Side view of the Gr/α-Al_2O_3 interface with additional H atom. H atom is on top of the surface Al atom, and binds to a C atom in the Gr layer. The additional H atom directly bond to part of the C atoms of Gr with a bond length of 1.1711 Å, but the other C atoms are further away from the interface. As a result, the average spacing between the additional H atom and the Gr is 1.4732 Å. The distance between the surface Al atom to additional H layer is 2.0642 Å, and the interlayer distance between the surface Al and the subsurface O layers is only 0.1978 Å. Obviously, such a structure is not consistent with the TEM images.

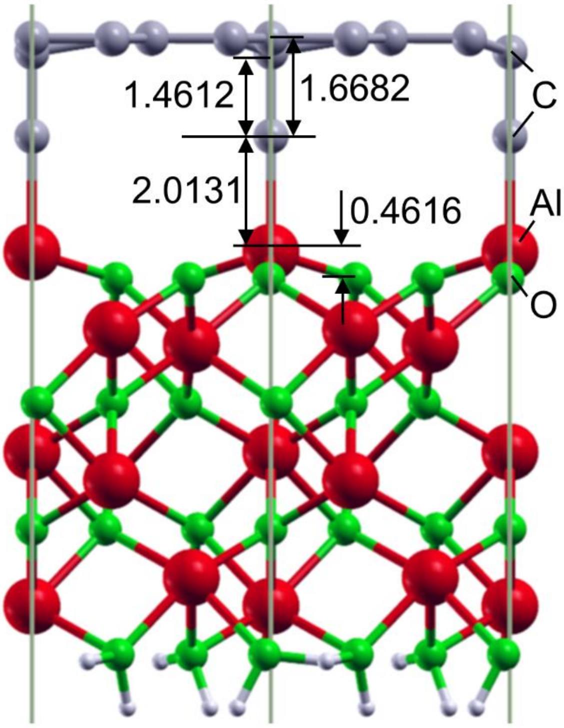

Supplementary Figure 5. DFT calculation of Gr/α-Al_2O_3 with C at the interface. Side view of the Gr/α-Al_2O_3 interface with additional C atom from DFT calculation. C atom is on top of the surface Al atom, and binds to a C atom in the Gr layer. The additional C atom directly bond to part of the C atoms of Gr with a bond length of 1.4612 Å, but the other C atoms are further away from the interface. As a result, the average spacing between the additional C atom and the Gr is 1.6682 Å. The distance between the surface Al atom to additional C layer is 2.0131 Å, and the interlayer distance between the surface Al and the subsurface O layers is 0.4616 Å. Obviously, such a structure is not consistent with the TEM images.

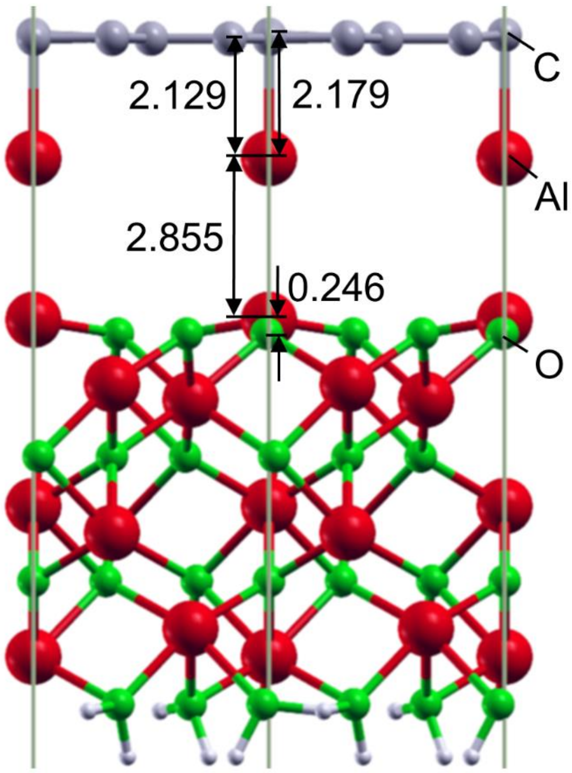

Supplementary Figure 6. DFT calculation of Gr/α-Al_2O_3 with Al at the interface. Side view of the Gr/α-Al_2O_3 interface with additional Al atom from DFT calculation. Al atom is on top of the surface Al atom, and binds to a C atom in the Gr layer. The additional Al atom directly bond to part of the C atoms of Gr with a bond length of 2.1788 Å, but the other C atoms are closer to the interface. As a result, the average spacing between the additional Al atom and the Gr is 2.1288 Å. The distance between the surface Al atom to additional Al layer is 2.8545 Å, and the interlayer distance between the surface Al and the subsurface O layers is 0.2459 Å. Obviously, such a structure is not consistent with the TEM images.

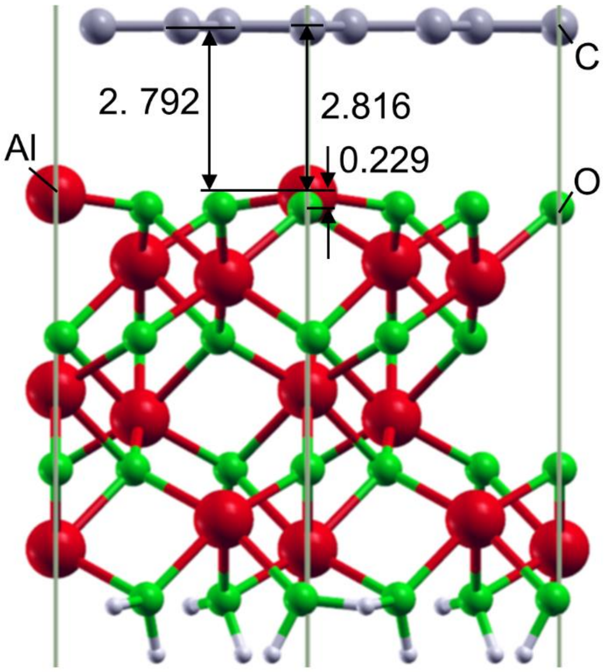

Supplementary Figure 7. DFT calculation of Gr/α-Al_2O_3 without interfacial atom. Side view of the Gr/α-Al_2O_3 interface from DFT calculation. No additional atom exists at the interface. The distance between the surface Al atom and the C atom on top of it is 2.7922 Å, and the other C atoms are slightly further away from the interface. As a result, the average spacing between the surface Al and the Gr is 2.8161 Å. The interlayer distance between the surface Al and the subsurface O layers is 0.2291 Å.

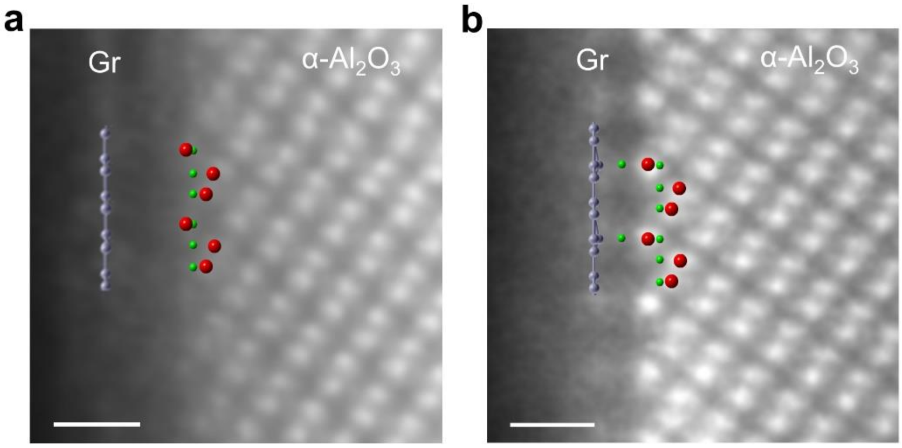

Supplementary Figure 8. Structure of Gr/α-Al_2O_3 interface. (a) The structure of transferred Gr/α-Al_2O_3 interface. (b) The structure of transferred Gr/α-Al_2O_3 interface after annealing. Red: Al atom. Green: O atom. Gray: C atom. Scale bar: 0.5 nm.

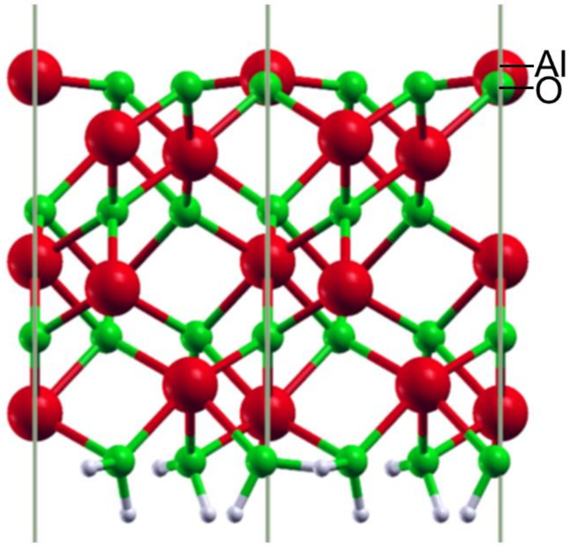

Supplementary Figure 9. The DFT calculation of α-Al_2O_3 surface structure.

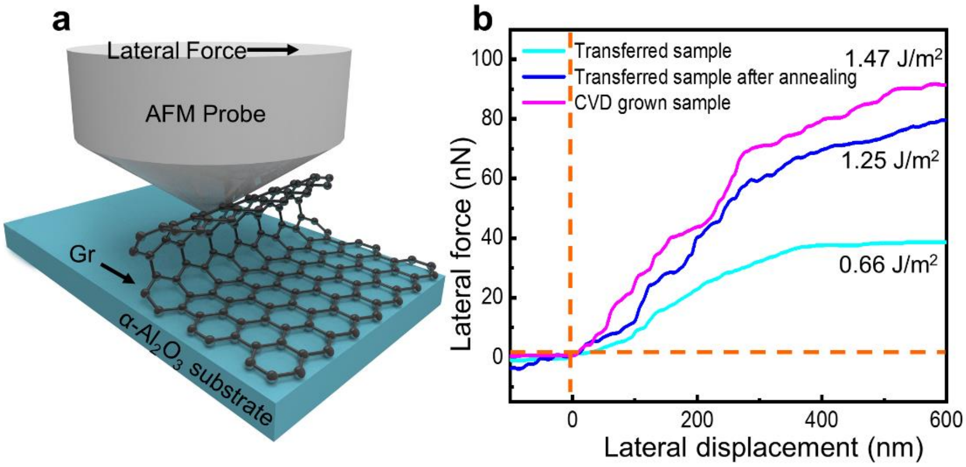

Supplementary Figure 10. Nano-scratch measurement. (a) Schematic representation of the nano-scratch method. (b) Lateral force versus displacement curves of directly grown, transferred, and post annealed Gr on α-Al_2O_3 based mechanical nano-scratch method.

Supplementary References

1 Kresse, G. & Hafner, J. Ab initio molecular dynamics for liquid metals. *Physical Review B* **47**, 558-561 (1993).

2 Perdew, J. P., Burke, K. & Ernzerhof, M. Generalized gradient approximation made simple. *Physical Review Letters* **77**, 3865-3868 (1996).

3 Blöchl, P. E. Projector augmented-wave method. *Physical Review B* **50**, 17953-17979 (1994).

4 Monkhorst, H. J. & Pack, J. D. Special points for Brillouin-zone integrations. *Physical Review B* **13**, 5188-5192 (1976).

5 Klimeš, J., Bowler, D. R. & Michaelides, A. Van der Waals density functionals applied to solids. *Physical Review B* **83**, 195131 (2011).
